# Evidence for startle as a measurable behavioral indicator of motor learning

**DOI:** 10.1371/journal.pone.0195689

**Published:** 2018-05-09

**Authors:** Nathan J. Kirkpatrick, Vengateswaran J. Ravichandran, Eric J. Perreault, Sydney Y. Schaefer, Claire F. Honeycutt

**Affiliations:** 1 School of Biological and Health Systems Engineering, Arizona State University, Tempe, AZ, United States of America; 2 Sensory Motor Performance Program, Rehabilitation Institute of Chicago, Chicago, IL, United States of America; 3 Department of Biomedical Engineering, Northwestern University, Evanston, IL, United States of America; 4 Department of Physical Medicine and Rehabilitation, Northwestern University, Chicago, IL, United States of America; Curtin University, AUSTRALIA

## Abstract

The ability of the classic startle reflex to evoke voluntarily prepared movement involuntarily has captured the attention of neuroscientists for its wide-ranging functional utility and potential uses in patient populations. To date, there is only one documented task resistant to the startReact phenomenon–index finger abduction. Previous reports have suggested the lack of startReact is due to different neural mechanisms driving individuated finger movement and more proximal joint control (e.g. elbow, wrist movement). However, an alternative hypothesis exists. Though not particularly difficult to execute, isolated index finger abduction is rarely performed during activities of daily living and is not a natural correlate to common individuated finger tasks. We propose that startReact can be evoked during individuated finger movements but only during tasks that are highly trained or familiar. The objective of this study was to determine the impact of a 2-week training regimen on the ability to elicit startReact. We found evidence in support of our hypothesis that following training, individuated movements of the hands (specifically index finger abduction) become susceptible to startReact. This is significant not only because it indicates that individuated finger movements are in fact amenable to startReact, but also that startle has differential response characteristics in novel tasks compared to highly trained tasks suggesting that startle is a measurable behavioral indicator of motor learning.

## Introduction

Discovered a mere 18 years ago, the ability of a startling acoustic stimulus to involuntarily evoke planned movement has captured the attention of neuroscientists for its wide-ranging functional utility and potential uses in patient populations. These movements, referred to as startReact, are easy to elicit through loud acoustic stimuli [1]. StartReact releases movement patterns which maintain the spatial and temporal characteristics of voluntary movements except that they are released at least 30 ms earlier–the result of an alternative release mechanism [2]. StartReact movements have been replicated by numerous groups across the globe and have become a mainstay in the literature due to their robustness across multiple joints (e.g. elbow, wrist, ankle), tasks (e.g. balance, position, force control), and populations (e.g. stroke, Parkinson’s disease, spinal cord injury; [1–11]). StartReact has been implicated in contributing to the reflexive response to maintain postural stability in the whole body and upper extremity [12–14]. StartReact movements have also been utilized as a probe of the brainstem and reticulospinal systems [1, 3, 4, 15]. Perhaps startReact’s most provocative outcome is its ability to enhance movements of stroke survivors [6, 11, 16].

To date, there has only been one documented task resistant to startReact–index finger abduction. While some groups have demonstrated that individuated finger movements exhibit startReact [17–19], two independent groups have shown that startReact is absent during index finger abduction [15, 20]. These authors have suggested the lack of startReact in index finger abduction is due to different neural mechanisms driving individuated finger movement and larger reaching movements using proximal joints. There is some evidence that startReact is mediated via the brainstem and reticulospinal pathways [1, 3–5, 21–25]. Therefore, tasks that rely heavily on corticospinal projections, such as isolated index finger abduction, would not generate startReact because those movements do not rely (or rely less) on these structures. However, the neural mechanisms governing startReact are contested [3–5, 15, 19, 26–29] and there is growing evidence that startling stimuli evoke a large cortical response [17, 30–32]. Furthermore, grasping movements of the hand that also use the first dorsal interosseous (FDI) muscle readily evoke startReact and arguably would utilize the same (or similar) neural structures [33].

Thus, we propose an alternative hypothesis–startReact is more accessible in familiar or highly trained tasks. Isolated index finger abduction, though not particularly difficult to perform for most, is rarely utilized during daily life [34]. An observation of activities of daily living found that the most common individuated finger movements were those that required interacting with objects (e.g. keys, writing elements). These tasks required thumb extension, combinations of thumb and individual other fingers, lateral pinch (i.e. key grip), and index finger extension [34]. Index finger abduction was not observed in the study, again highlighting its uncommon usage.

We propose that startReact can be more readily evoked during individuated finger movements that are highly trained or familiar. The objective of this study was to determine whether extensive practice of an individuated finger movement (specifically index finger abduction) would impact the ability to elicit startReact. We hypothesized that following two weeks of training, index finger abduction would more readily evoke startReact. This would be significant not only because it would indicate that individuated finger movements are in fact amenable to startReact, but also that startReact has differential response characteristics in novel tasks compared to highly trained tasks, suggesting startle and startReact may be a measurable behavioral indicator of motor learning.

## Methods

### Subjects

Nine right-hand dominant subjects (2 males, 7 females; age: 22.5 ± 2.6 years) qualified to take part in this 10-day training study and were included in final data analysis. These subjects met inclusion criteria of: no apparent physical abnormalities, no sensory or motor dysfunctions, no surgeries or injuries to hands or upper extremities, no hearing loss or sensitivity, no heart conditions, not currently pregnant, and absence of startReact in index finger abduction on Day 1. Five participants did not qualify for the study because they exhibited startReact on the first day. Before experimentation, a detailed explanation of procedures and risks was provided to all subjects and express written consent for participation was obtained in accordance with the provisions set forth by the Institutional Review Boards of Arizona State University (MOD00003436) and Northwestern University (STU9204).

### Equipment and setup

Electromyography (EMG) was recorded from the right and left sternocleidomastoid muscles (RSCM, LSCM) and the right FDI muscle with bipolar electrodes (solid gel, Ag-AgCl surface electrode; MVAP Medical Supplies, Newbury Park, CA). Electrodes were placed on the belly of the FDI, RSCM and LSCM muscles, with a unipolar ground electrode (solid gel, Ag-AgCl surface electrode; MVAP Medical Supplies, Newbury Park, CA) over the right ulnar styloid process. Preamplifiers (model no. AMT-8; 500 gain; Bortec, Calgary, Alberta, Canada) with a band-pass filter of 10–1,000 Hz were used on the EMG data.

### Experimental design

All protocols have been previously described [15]. Briefly, subjects were seated in a comfortable, height-adjustable chair. The right hand and arm rested comfortably on an arm rest at an elbow angle of approximately 90 degrees. Forearms were restrained with padded straps to minimize motion during the experiment.

**Fig 1:**
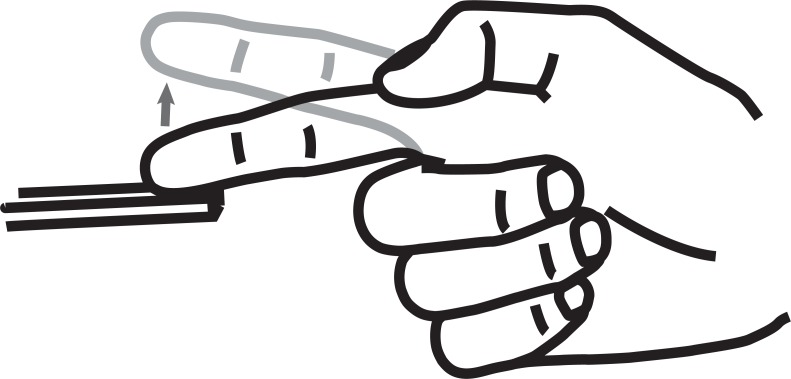
Task illustration. Subjects performed index finger abduction with their right hand when directed by the auditory GO cue.

Subjects completed an index finger abduction task where the finger moved from a relaxed position towards the ceiling (Fig 1). Task completion was validated with a switch device (D2VW-5L1B-3HS; Omron) oriented to be depressed when the subject’s index finger was at rest. Switch height and angle were adjusted for each subject to allow for the index finger to point straight along the axis of the forearm and to be free to move.

Subjects were instructed to prepare to move corresponding to two auditory sounds: READY and GO. They were instructed to complete the index finger abduction task as quickly as possible after the GO. The time interval between these READY and GO sounds was randomized between 1.5 and 3.5 sec to prevent anticipation of the GO signal in accordance with previously published protocols [15].

Experiments consisted of 2 different types of trials: Stimulus and No Stimulus trials. During No Stimulus trials, both auditory sounds were presented at a soft, 80 dB via computer speakers. During the Stimulus trials, the 80 dB GO was replaced with a loud, startling acoustic stimulus of 120 dB. This occurred randomly in one third of trials and was never presented consecutively. The loud startling sounds were presented via a loud horn (TS-333S Siren Speaker, Chowsons International Inc.) located approximately 35 cm behind the subject’s head. The decibel was tested at the beginning of each experiment with a decibel reader (SSEYL AZ-8928 Digital Sound Level Meter, AZ Instrument). The loud stimulus lasted 25 ms and had a rise time of 0.33 ms.

**Fig 2:**
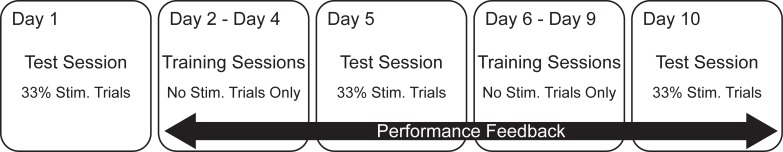
Experiment schedule. Subjects practiced index finger abduction over 10 daily training sessions. Stimulus trials were included on Day 1, 5 and 10. Auditory feedback based on previous day’s performance was administered on Day 2–10.

Experiments were split into test sessions (3 sessions) and training sessions (7 sessions; Fig 2). Test sessions were completed on Day 1, 5 and 10 and comprised an average of 10 blocks of 15 trials (Fig 2). During these test sessions, subjects first practiced the task in one block of 15 No Stimulus trials before performing the task in blocks containing Stimulus trials. Training sessions were held on days 2–4 and 6–9 and featured only No Stimulus trials in 6 blocks of 15 trials.

To encourage attention and learning, auditory performance feedback was provided to the subjects after each No Stimulus trial on Day 2–10 in the form of two distinct two-tone sounds played on computer speakers (60 dB). Before the start of the first training session, subjects were instructed to treat one sound as positive feedback, and the other as negative feedback. Positive or negative acoustic feedback was delivered based on a comparison between a trial’s completion time and that of the previous day’s average No Stimulus task completion time. Completion times for performance feedback was determined as the time between the GO cue and when the switch device changed state from depressed to un-depressed.

### Data analysis

All EMG data were processed in MATLAB (R2014b; MathWorks). FDI muscle onset latency was calculated for each trial after the DC offset was removed, and the signal was rectified and smoothed with a 10-point moving average filter. A conservative automated program determined when the processed EMG signal remained above three times the standard deviation of the previous 500 ms for at least 5 ms for the FDI signal (onset latency, [15]). Each identified onset latency was visually evaluated to ensure accuracy by a reviewer blinded to both trial type and day.

### StartReact

We hypothesized index finger abduction would be more susceptible to startReact following motor training. Across all subjects, a total of 1181 Stimulus trials were analyzed. To determine if a task is susceptible to startReact, the intensity-dependent and startle effects must be evaluated separately. First, not all trials with a loud, startlingly sound elicit a startReact–much like not all loud sounds cause a classic startle or “flinching” response. In order to detect the presence of a startle, SCM activity was monitored [35]. When a task is susceptible to startReact, the presence of the startle reflex (quantified by activity in the SCM muscle) decreases the onset latency. However, onset latencies also decrease in response to increasing auditory stimulus intensities (i.e. subjects react more quickly when the GO stimulus is a louder sound or brighter light [36, 37]). To differentiate between these two factors, it is necessary to compare trials with and without SCM activity during Stimulus trials only. Comparing Stimulus trials directly to No Stimulus trials without monitoring SCM can lead to “false positives” (i.e. trials are considered to exhibit startReact due to intensity-level effects and not due to the influence of startle). This methodology has been established in the literature [2, 38] and remains the only method (to our knowledge) to allow the separation of intensity and startle-related effects on onset latency.

Trials with SCM activity are denoted SCM+ and those without SCM activity were classified SCM-. A task is considered to evoke startReact if SCM+ trials demonstrate faster onset latencies compared to SCM-. Trials with abnormal SCM EMG activity and/or irregular background activity were excluded from further analysis. Less than 2.5% of trials were excluded based on these criteria.

It is worth noting that in some tasks SCM maybe an unreliable measure of startle [13, 39]. Specifically, classification based on SCM in tasks where neck musculature is actived during the normal execution of the task (e.g. a perturbation of the arm or large reaching movements) could prove inaccurate. Indeed, authors evaluating these tasks have indicated that SCM is activated during No Stimulus trials or during the normal execution of the task [13, 39]. In our study, SCM was almost never (0.8%) present during No Stimulus trials and therefore remains a viable method to evaluate the presence of startle.

### Statistical analyses

We expected FDI movement would not elicit startReact on Day 1 (confirming previous reports; [15, 20]), but would develop by Day 10, indicated by a difference between SCM+ and SCM- trials. For Day 1, we hypothesized that the FDI onset latency would be equivalent between SCM+ and SCM- trials. Therefore, two one-sided T-tests (TOST) were used to test the null hypothesis that the SCM+ and SCM- latencies were different. The TOSTER package in R was executed. As is convention for TOST, we utilized a 90% confidence interval (5% for each side). We conservatively used the smaller standard deviation of the two distributions as the equivalence bound. For Days 5 and 10, we hypothesized that the means would be different. Therefore, a univariate, linear mixed-effects model was applied to the FDI onset latency with the condition (SCM+ or SCM-) and day as the independent factors, and subject as a random factor, using SPSS (SPSS 23; IBM). To account for Type I error as a result of unbalanced data designs inherent in startReact, as well as unequal variance in within-day condition sample groupings, an α of 0.01 was used [40]. Two subjects were excluded because of a lack of at least two SCM+ and SCM- trials during each test session and are not included in the 9 qualifying subjects described above.

To verify motor learning, No Stimulus trials during test sessions were identified and the time between the GO cue and EMG onset was calculated for each (onset latency; [41]). Learning was assessed by a repeated measures ANOVA of FDI EMG onset latency with subject as a random factor in SPSS amongst No Stimulus trials on Days 1, 5 and 10. Tukey’s Honestly Significant Difference (TukeyHSD) was applied for post-hoc comparisons between days. We expected No Stimulus onset latencies would decrease in response to the training regimen.

Probability of eliciting a startle was calculated as the percentage of SCM+ trials to all stimulus trials for each subject and for each test session. The Friedman ranked sum test, which is a non-parametric test, was used to evaluate differences in the probability of startle across different days (i.e. matched condition) regardless of the distribution.

## Results

**Fig 3:**
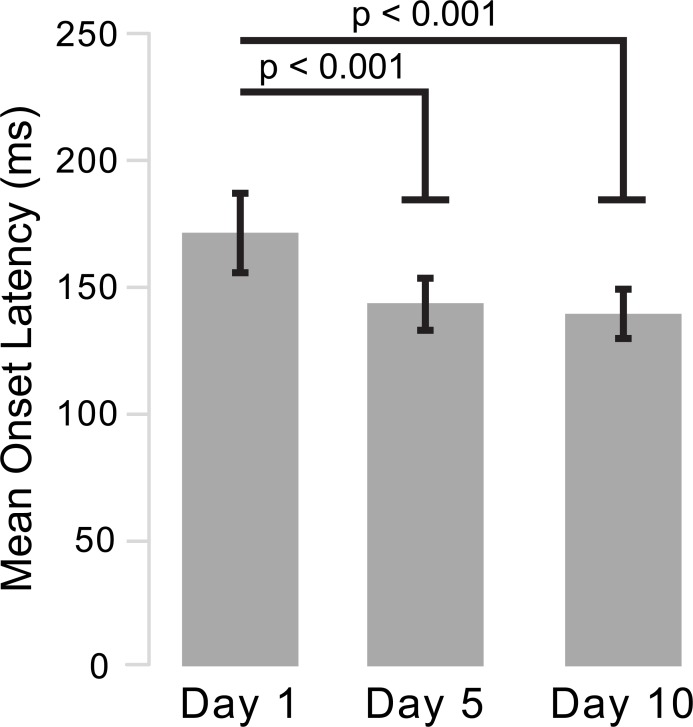
Voluntary onset latencies. Average FDI EMG onset latencies and standard errors during No Stimulus trials on Days 1, 5, and 10 are depicted to highlight that training occurred.

Onset latencies during No Stimulus trials decreased from the first to the last day of training, indicating motor learning occurred (Fig 3; *F*_*2*,*2286*_ = 130.0, p < 0.001). FDI onset latencies for No stimulus trials decreased from Day 1 (171.4 ± 48.2 ms) to Day 5 (142.9 ± 31.3 ms, p < 0.001), and Day 1 to Day 10 (139.2 ± 30.3 ms, p < 0.001). Though a difference was found between Day 5 to Day 10, it did not reach significance (Δ = 3.7 ms, p = 0.09).

**Fig 4:**
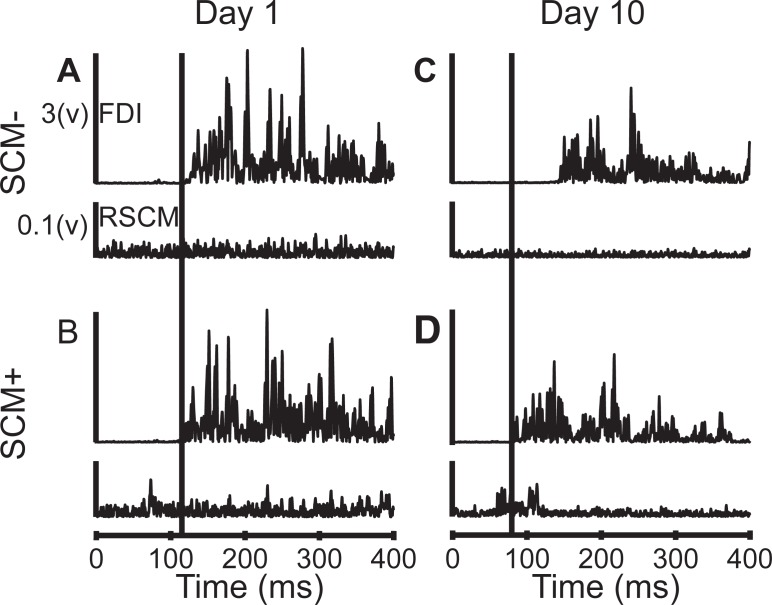
Sample EMG data from SCM+ and SCM- trials. *A* & *B*: EMG data from FDI and RSCM muscles during SCM+ and SCM- trials acquired on Day 1. *C* & *D*: FDI and RSCM EMG data during SCM+ and SCM- trials acquired on Day 10. Vertical lines mark FDI onset of SCM+ for comparison to SCM-.

The presence of startle (SCM+) did not influence FDI onset latency on Days 1 and 5 but showed faster latencies by Day 10. The presence of startle (SCM+) on Day 1 did not influence FDI onset latency (Fig 4A and 4B) with SCM+ (108.4 ± 2.3 ms) and SCM- (107.4 ± 1.9 ms) reporting similar latencies. On Day 5, SCM+ trials were faster (100.9 ± 4.1 ms) than SCM- trials (107.1 ± 3.7 ms). Finger abduction was susceptible to startReact on Day 10. FDI onset latency was faster when startle was present (SCM+: 92.3 ± 4.3 ms) compared to when startle was absent (SCM-: 101.0 ± 2.7 ms, Fig 4C and 4D).

**Fig 5:**
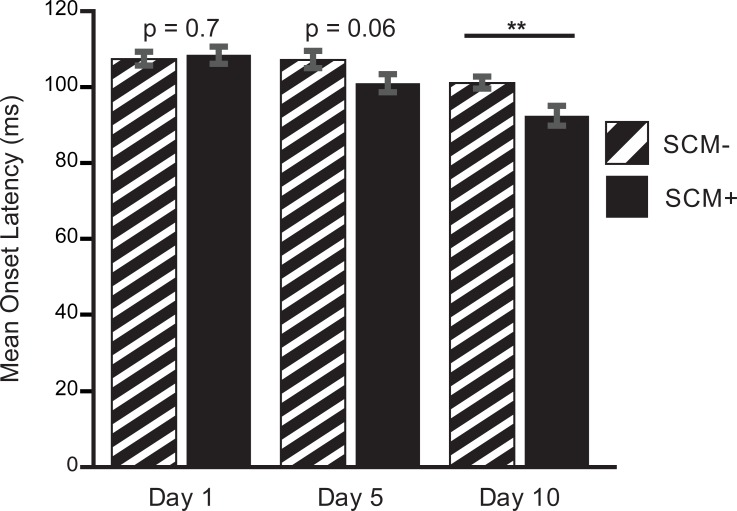
Group results. Comparisons of FDI onset latencies between SCM+ and SCM- trials. **P < 0.01, SE error bars.

Group results confirmed that the presence of startle (SCM+) did not influence finger abduction latency on Days 1 and 5 but developed by Day 10. There were significant main effects on FDI onset latency for day (*F*_*2*,*1097*_ = 13.979, p < 0.001) as well as presence of startle (*F*_*1*,*1097*_ = 6.617, p = 0.010). Pairwise comparisons of startle presence within each day of training supported a lack of difference on Day 1 (Fig 5; Δ = -1.0 ms; p = 0.7). Day 5 saw a larger difference between SCM+/SCM- trial comparisons but it did not reach significance (Fig 5; Δ = 6.3 ms, p = 0.06). By Day 10, startReact was present (Fig 5; Δ = 8.7 ms, p = 0.004). On Day 1, TOST test rejected the null hypothesis that SCM+ and SCM- onset latencies were different (t_1149_ = -7.076, p < 0.001); further confirming that SCM+ and SCM- latencies were equivalent on Day 1.

The differences between SCM+ and SCM- trials across the training program were not the result of a change in startle excitability. The percentage of SCM+ trials was not different between Day 1 (42.0 ± 28.8%), Day 5 (39.9 ± 32.7%), and Day 10 (37.3 ± 26.1%) (P = 0.3679; Chi-squared = 2, df = 2).

## Discussion

The objective of this study was to determine the impact of a 2-week training regimen on the ability to elicit startReact. We hypothesized that following training, individuated movements of the hands (specifically index finger abduction) would be susceptible to startReact. We indeed found that startReact was not present on Day 1 but was fully present on Day 10. Our study is the first to show that startReact is accessible in individuated movements of the fingers, which has implications in its use as a therapy tool. Perhaps more interestingly, startReact is only accessible in highly practiced or trained tasks suggesting that startReact is a measurable behavior indicator for motor learning.

### StartReact is present in individuated finger movements

StartReact is remarkably robust across multiple joints (e.g. elbow, wrist, ankle), tasks (e.g. position tasks, multi-phased movements), and patient populations (e.g. stroke, Parkinson’s disease, Spinal cord injury; [1–11]); however, its presence in individuated finger movements has been questioned. While some groups have demonstrated that individuated finger movements are susceptible to startReact [17–19], others have shown that index finger abduction is resistant to startReact [15, 20]. The data presented here agrees with the former studies and indicates that individuated finger movements are readily susceptible to startReact under certain conditions.

Those studies that found a lack of startReact evaluated upward index finger abduction, which is an individuated finger movement but is a poor correlate for everyday hand function. An observation of activities of daily living found that the most common individuated finger movements were those that required interacting with objects (e.g. keys, writing elements). These tasks required thumb extension, combinations of thumb and individual other fingers, lateral pinch (i.e. key grip), and index finger extension [34]. Index finger abduction was not observed in the study, again highlighting its uncommon utilization.

This study showing that startReact is present during index finger abduction after training indicates that startReact does not exist within a proximal-distal gradient but rather is defined by task familiarity. To date, most tasks that have been evaluated with startle are simple, common tasks that are analogs to reaching. Our work highlights that individuated movements of the distal limb may be readily accessible via startReact provided they are also commonly performed. For example, hand flexion and extension are readily accessible via startReact. Though the primary agonists for hand extension and flexion are more proximal in the forearm, full execution of the task requires extension and flexion of the intrinsic muscles of the fingers and hand. Future work should assess common tasks of daily living (e.g. object manipulation) to determine the extent to which these movements are accessible to startReact. We conclude that the full repertoire of movements that are accessible via startReact includes individuated finger movements.

### Startle as a behavioral indicator of motor learning

Numerous studies have highlighted a shift in the neural structures used over the course of motor learning. Motor learning, the process by which movements are honed and refined to become faster and more accurate [42], utilizes a diverse set of neural structures including the cortex [43–47], subcortex [48], cerebellum [49], and brainstem [50, 51]. However, it is also clear that the utilization of these structures changes following extensive training. For example, a recent study published in Neuron demonstrated that there is a transfer from cortical to subcortical structures following intense training [52]. Thus, the emergence of startReact at the end of training, but not at the beginning of training, likely corresponds to a shift in the neural structures mediating motor planning between novel and trained tasks. While some studies suggest startReact is a probe of the brainstem and reticulospinal system [3–5, 15, 19, 26–29], others have shown that, similar to the stretch reflex, a cortical loop may be present [17, 30, 31], leaving the neural mechanisms mediating startReact somewhat controversial. As the neural structures governing startReact are elucidated, this report lays the foundation for startReact to be used as a non-invasive probe of motor skill acquisition in both healthy and impaired populations.

While startReact detects a shift from novel to trained, it is important to note that startReact does not appear to detect more subtle shifts in learning if the task is already familiar. Training has no effect on the startReact response [53] in tasks that already exhibit a robust startReact response (wrist extension/flexion). This indicates that a task must be sufficiently novel for startReact to detect shifts in learning.

### Clinical ramifications

We have previously demonstrated that startReact can significantly enhance the movement performance of elbow and hand movements in stroke survivors [6, 11, 54]. Although voluntary movement onset latencies for stroke survivors were delayed compared to age-matched unimpaired subjects, the presence of startle eliminated the differences between the populations for ballistic elbow movements as well as fast hand extension. The functional relevance of these improvements is unknown at this time; however, the results here indicate that startReact is accessible even in individuated movements of the fingers and therefore startReact may be able to significantly enhance movement during dexterous object manipulation (provided that people have learned the task prior to their stroke) which are often the most significantly disabled movements following stroke.

## Conclusions

Individuated finger movements are susceptible to startReact following a 10-day training regimen. This indicates that startReact has differential effects between novel and highly trained tasks, making it a natural non-invasive probe of skilled motor acquisition in healthy and impaired populations.

## Supporting information

S1 File EMG onset latency and percentage of startle. Means and standard deviations for all subjects for Stimulus and No Stimulus trials are presented. The following characteristics of the data are specified: presence of startle (SCM+ or SCM-), Day (1, 5, 10), and number of trials (N). Finally, the percentage of Stimulus trials that resulted in a startle (SCM+) are presented for all subjects and Days.
(XLSX)

## References

[1] Valls-Solé J, Rothwell JC, Goulart F, Muñoz E, Cossu G. Patterned ballistic movements triggered by a startle in healthy humans. J Physiol. 1999; 516 (Pt 3:931–8. doi: 10.1111/j.1469-7793.1999.0931u.x 10200438 10.1111/j.1469-7793.1999.0931u.x PMC2269293

[2] Carlsen AN, Maslovat D, Lam MY, Chua R, Franks IM. Considerations for the use of a startling acoustic stimulus in studies of motor preparation in humans. Neuroscience and biobehavioral reviews. 2011; 35(3):366–76. doi: 10.1016/j.neubiorev.2010.04.009 PubMed PMID: ISI:000286639900003. 20466020 10.1016/j.neubiorev.2010.04.009

[3] Carlsen AN, Chua R, Inglis JT, Sanderson DJ, Franks IM. Can prepared responses be stored subcortically? Exp Brain Res. 2004; 159(3):301–9. doi: 10.1007/s00221-004-1924-z . 15480608 10.1007/s00221-004-1924-z

[4] Carlsen AN, Chua R, Inglis JT, Sanderson DJ, Franks IM. Prepared movements are elicited early by startle. Journal of Motor Behavior. 2004; 36:253–64. doi: 10.3200/JMBR.36.3.253-264 15262622 10.3200/JMBR.36.3.253-264

[5] Nonnekes J, Oude Nijhuis LB, de Niet M, de Bot ST, Pasman JW, van de Warrenburg BP, et al StartReact restores reaction time in HSP: evidence for subcortical release of a motor program. J Neurosci. 2014; 34(1):275–81. doi: 10.1523/JNEUROSCI.2948-13.2014 . 24381288 10.1523/JNEUROSCI.2948-13.2014 PMC6608175

[6] Honeycutt CF, Tresch UA, Perreault EJ. Startling acoustic stimuli can evoke fast hand extension movements in stroke survivors. Clin Neurophysiol. 2014; doi: 10.1016/j.clinph.2014.05.025 . 25002367 10.1016/j.clinph.2014.05.025 PMC4268121

[7] Nonnekes J, Geurts AC, Oude Nijhuis LB, van Geel K, Snijders AH, Bloem BR, et al Reduced StartReact effect and freezing of gait in Parkinson's disease: two of a kind? Journal of neurology. 2014; doi: 10.1007/s00415-014-7304-0 . 24609973 10.1007/s00415-014-7304-0

[8] Jankelowitz SK, Colebatch JG. The acoustic startle reflex in ischemic stroke. Neurology. 2004; 62:114–6. 14718710 10.1212/01.wnl.0000101711.48946.35

[9] Cressman EK, Carlsen AN, Chua R, Franks IM. Temporal uncertainty does not affect response latencies of movements produced during startle reactions. Experimental brain research. 2006; 171:278–82. doi: 10.1007/s00221-006-0459-x . 16604311 10.1007/s00221-006-0459-x

[10] Siegmund GP, Inglis JT, Sanderson DJ. Startle response of human neck muscles sculpted by readiness to perform ballistic head movements. The Journal of physiology. 2001; 535:289–300. doi: 10.1111/j.1469-7793.2001.00289.x 11507178 10.1111/j.1469-7793.2001.00289.x PMC2278755

[11] Honeycutt CF, Perreault EJ. Planning of ballistic movement following stroke insights from the startle reflex. PLoS One. 2012; 7(8):1–11. doi: 10.1371/journal.pone.0043097.t001 10.1371/journal.pone.0043097 PMC3431358 22952634

[12] Campbell AD, Chua R, Inglis JT, Carpenter MG. Startle induces early initiation of classically conditioned postural responses. J Neurophysiol. 2012; 108(11):2946–56. doi: 10.1152/jn.01157.2011 . 22972964 10.1152/jn.01157.2011

[13] Forgaard CJ, Franks IM, Maslovat D, Gowan NJ, Kim JC, Chua R. An examination of the startle response during upper limb stretch perturbations. Neuroscience. 2016; 337:163–76. doi: 10.1016/j.neuroscience.2016.09.010 . 27664458 10.1016/j.neuroscience.2016.09.010

[14] Ravichandran VJ, Honeycutt CF, Shemmell J, Perreault EJ. Instruction-dependent modulation of the long-latency stretch reflex is associated with indicators of startle. Exp Brain Res. 2013; 230(1):59–69. doi: 10.1007/s00221-013-3630-1 ; PubMed Central PMCID: PMCPMC3759548. 23811739 10.1007/s00221-013-3630-1 PMC3759548

[15] Honeycutt CF, Kharouta M, Perreault EJ. Evidence for reticulospinal contributions to coordinated finger movements in humans. J Neurophysiol. 2013; 110(7):1476–83. doi: 10.1152/jn.00866.2012 ; PubMed Central PMCID: PMCPMC4042417. 23825395 10.1152/jn.00866.2012 PMC4042417

[16] Marinovic W, Brauer SG, Hayward KS, Carroll TJ, Riek S. Electric and acoustic stimulation during movement preparation can facilitate movement execution in healthy participants and stroke survivors. Neurosci Lett. 2016; 618:134–138. doi: 10.1016/j.neulet.2016.03.009 26964472 10.1016/j.neulet.2016.03.009

[17] Marinovic W, Tresilian JR, Rugy AD, Sidhu S, Riek S. Corticospinal modulation induced by sounds depends on action preparedness. The Journal of Physiology. 2013; 592: 153–169. doi: 10.1113/jphysiol.2013.254581 24081157 10.1113/jphysiol.2013.254581 PMC3903357

[18] Marinovic W, Cheung FLY, Riek S, Tresilian JR. The effect of attention on the release of anticipatory timing actions. Behavioral Neuroscience. 2014; 128: 548–555. doi: 10.1037/bne0000007 25150545 10.1037/bne0000007

[19] Tazoe T, Perez MA. Cortical and reticular contributions to human precision and power grip. The Journal of Physiology. 2017; 595: 2715–2730. doi: 10.1113/JP273679 27891607 10.1113/JP273679 PMC5390869

[20] Carlsen AN, Chua R, Inglis JT, Sanderson DJ, Franks IM. Differential effects of startle on reaction time for finger and arm movements. J Neurophysiol. 2008; 101(1):306–14. doi: 10.1152/jn.00878.2007 ; PubMed Central PMCID: PMCPMC2637008. 19005006 10.1152/jn.00878.2007 PMC2637008

[21] Hammond GR. Lesions of pontine and medullary reticular formation and prestimulus inhibition of the acoustic startle reaction in rat. Physiology and Behavior. 1973; 10:239–43. 4575307 10.1016/0031-9384(73)90304-1

[22] Groves PM, Wilson CJ, Boyle RD. Brain stem pathways, cortical modulation, and habituation of the acoustic startle response. Behavioral Biology. 1974; 10:391–418. 4832941 10.1016/s0091-6773(74)91975-0

[23] Davis M, Gendelman PM. Plasticity of the acoustic startle response in the acutely decerebrate rat. Journal of Comparative and Physiological Psychology. 1977; 91(3):549–63. 874121 10.1037/h0077345

[24] Davis M, Gendelman DS, Tischler MD, Gendelman PM. A primary acoustic startle circuit lesion and stimulation studies. The Journal of Neuroscience. 1982; 2(6):791–805. 7086484 10.1523/JNEUROSCI.02-06-00791.1982 PMC6564345

[25] Rothwell JC. Chapter 18 The startle reflex, voluntary movement, and the reticulospinal tract. Supplements to Clinical Neurophysiology Brainstem Function and Dysfunction. 2006; 223–31. doi: 10.1016/s1567-424x(09)70071-6 10.1016/s1567-424x(09)70071-6 16623334

[26] Nonnekes J, de Niet M, Oude Nijhuis LB, de Bot ST, van de Warrenburg BP, Bloem BR, et al Mechanisms of postural instability in hereditary spastic paraplegia. J Neurol. 2013; 260(9):2387–95. doi: 10.1007/s00415-013-7002-3 . 23784609 10.1007/s00415-013-7002-3

[27] Valls-Solé J. Chapter 58 Contribution of subcortical motor pathways to the execution of ballistic movements. Advances in Clinical Neurophysiology, Proceedings of the 27th International Congress of Clinical Neurophysiology, AAEM 50th Anniversary and 57th Annual Meeting of the ACNS Joint Meeting Supplements to Clinical Neurophysiology. 2004; 554–562. doi: 10.1016/s1567-424x(09)70394-0 10.1016/s1567-424x(09)70394-0 16106656

[28] Rothwell J, Mackinnon C, Valls-Solé J. Role of brainstem-spinal projections in voluntary movement. Movement Disorders. 2002; 17 doi: 10.1002/mds.10054 11836749 10.1002/mds.10054

[29] Baker SN, Perez MA. Reticulospinal Contributions to Gross Hand Function after Human Spinal Cord Injury. The Journal of Neuroscience. 2017; 37: 9778–9784. doi: 10.1523/JNEUROSCI.3368-16.2017 28871033 10.1523/JNEUROSCI.3368-16.2017 PMC5628413

[30] Marinovic W, Flannery V, Riek S. The effects of preparation and acoustic stimulation on contralateral and ipsilateral corticospinal excitability. Human Movement Science. 2015; 42: 81–88. doi: 10.1016/j.humov.2015.05.003 25988845 10.1016/j.humov.2015.05.003

[31] Alibiglou L, MacKinnon CD. The early release of planned movement by acoustic startle can be delayed by transcranial magnetic stimulation over the motor cortex. J Physiol. 2012; 590(4):919–36. doi: 10.1113/jphysiol.2011.219592 ; PubMed Central PMCID: PMCPMC3381319. 22124142 10.1113/jphysiol.2011.219592 PMC3381319

[32] Chen Y-T, Li S, Zhou P, Li S. Different Effects of Startling Acoustic Stimuli (SAS) on TMS-Induced Responses at Rest and during Sustained Voluntary Contraction. Frontiers in Human Neuroscience. 2016; 10 doi: 10.3389/fnhum.2016.00396 27547181 10.3389/fnhum.2016.00396 PMC4974269

[33] Baker SN. The primate reticulospinal tract, hand function and functional recovery. J Physiol. 2011; 589(Pt 23):5603–12. doi: 10.1113/jphysiol.2011.215160 ; PubMed Central PMCID: PMCPMC3249036 21878519 10.1113/jphysiol.2011.215160 PMC3249036

[34] Zheng JZ, Rosa SDL, Dollar AM. An investigation of grasp type and frequency in daily household and machine shop tasks. 2011 IEEE International Conference on Robotics and Automation. 2011; doi: 10.1109/icra.2011.5980366

[35] Brown P, Rothwell JC, Thompson PD, Britton TC, Day BL, Marsden CD. New observations on the normal auditory startle reflex in man. Brain Res. 1991; 114:1891–902. 10.1093/brain/114.4.1891 1884184

[36] Kohfeld DL. Effects of the intesity of auditory and visual ready-signals on simple reaction time. Journal of Experimental Psychology. 1969; 82(1):88–95. 5371997 10.1037/h0028033

[37] Kohfeld DL. Simple reaction time as a function of stimulus intensity in decibels of light and sound. Journal of Experimental Psychology. 1971; 88(2):251–7. 5577177 10.1037/h0030891

[38] Carlsen AN, Maslovat D, Franks IM. Preparation for voluntary movement in healthy and clinical populations Evidence from startle. Clinical Neurophysiology. 2012; 123:21–33. doi: 10.1016/j.clinph.2011.04.028 22033029 10.1016/j.clinph.2011.04.028

[39] Dean LR, Baker SN. Fractionation of muscle activity in rapid responses to startling cues. Journal of Neurophysiology. 2017; 117: 1713–1719. doi: 10.1152/jn.01009.2015 28003416 10.1152/jn.01009.2015 PMC5384977

[40] Norton DW. An empirical investigation of some effects of non-normality and heterogeneity on the F-distribution University of Iowa 1952.

[41] Laubach M, Wessberg J, Nicolelis MAL. Cortical ensemble activity increasingly predicts behaviour outcomes during learning of a motor task. Nature. 2000; 405:567–71. doi: 10.1038/35014604 10850715 10.1038/35014604

[42] Willingham DB. A neuropsychological theory of motor skill learning. Psychological Review. 1998; 105(3):558–84. 9697430 10.1037/0033-295x.105.3.558

[43] Petrides M. Deficits on conditional associative-learning tasks after frontal- and temporal-lobe lesions in man. Neuropsychologia. 1985; 23(5):601–14. 4058706 10.1016/0028-3932(85)90062-4

[44] Canavan AGM, Passingham RE, Marsden CD, Quinn N, Wyke M, Polkey CE. Prism adaptation and other tasks involving spatial abilities in patients with parkinsons-disease, patients with unilateral temporal lobectomies. Neuropsychologia. 1990; 28(9):969–84. 2259427 10.1016/0028-3932(90)90112-2

[45] Seitz RJ, Roland PE, Bohm C, Greitz T, Stone-Elander S. Motor learning in man a positron emission tomographic study. NeuroReport. 1990; 1:57–66. 2129858 10.1097/00001756-199009000-00016

[46] Grafton ST, Mazziotta JC, Presty S, Friston KJ, Frackowiak RSJ, Phelps ME. Functional anatomy of human procedural learning determined with regional cerebral blood flow and PET. The Journal of Neuroscience. 1992; 12(7):2542–8. 1613546 10.1523/JNEUROSCI.12-07-02542.1992 PMC6575851

[47] Muellbacher W, Ziemann U, Wissel J, Dang N, Kofler M, Facchini S, et al Early consolidation in human primary motor cortex. Nature. 2002; 415:640–4. doi: 10.1038/nature712 11807497 10.1038/nature712

[48] Heindel WC, Salmon DP, Shults CW, Walicke PA, Butters N. Neuropsychological evidence for multiple implicit memory systems: a comparison of Alzheimer's, Huntington’s, and Parkinson’s Disease. The Journal of Neuroscience. 1989; 9(2):582–7. 2521896 10.1523/JNEUROSCI.09-02-00582.1989 PMC6569801

[49] Weiner MJ, Hallett M, Funkenstein HH. Adaptation to lateral displacement of vision in patients with lesions of the central nervous system. Neurology. 1983; 33:766–72. 6682520 10.1212/wnl.33.6.766

[50] Bretzner F, Brownstone RM. Lhx3-Chx10 reticulospinal neurons in locomotor circuits. J Neurosci. 2013; 33(37):14681–92. doi: 10.1523/JNEUROSCI.5231-12.2013 . 24027269 10.1523/JNEUROSCI.5231-12.2013 PMC6705172

[51] Brownstone RM, Bui TV, Stifani N. Spinal circuits for motor learning. Curr Opin Neurobiol. 2015; 33:166–73. doi: 10.1016/j.conb.2015.04.007 . 25978563 10.1016/j.conb.2015.04.007

[52] Kawai R, Markman T, Poddar R, Ko R, Fantana AL, Dhawale AK, et al Motor cortex is required for learning but not for executing a motor skill. Neuron. 2015; 86(3):800–12. doi: 10.1016/j.neuron.2015.03.024 . 25892304 10.1016/j.neuron.2015.03.024 PMC5939934

[53] Maslovat D, Hodges NJ, Chua R, Franks IM. Motor preparation and the effects of practice: Evidence from startle. Behavioral Neuroscience. 2011; 125: 226–240. doi: 10.1037/a0022567 21280935 10.1037/a0022567

[54] Honeycutt CF, Perreault EJ. Deficits in startle-evoked arm movements increase with impairment following stroke. Clin Neurophysiol. 2014; 125(8):1682–8. doi: 10.1016/j.clinph.2013.12.102 ; PubMed Central PMCID: PMCPMC4076376. 24411525 10.1016/j.clinph.2013.12.102 PMC4076376

